# Copper and Melanin Play a Role in *Myxococcus xanthus* Predation on *Sinorhizobium meliloti*

**DOI:** 10.3389/fmicb.2020.00094

**Published:** 2020-02-04

**Authors:** Francisco Javier Contreras-Moreno, José Muñoz-Dorado, Natalia Isabel García-Tomsig, Gonzalo Martínez-Navajas, Juana Pérez, Aurelio Moraleda-Muñoz

**Affiliations:** ^1^Departamento de Microbiología, Facultad de Ciencias, Universidad de Granada, Granada, Spain; ^2^Estación Experimental del Zaidín, Granada, Spain

**Keywords:** *Myxococcus xanthus*, predation, copper, melanin, *Sinorhizobium meliloti*

## Abstract

*Myxococcus xanthus* is a soil myxobacterium that exhibits a complex lifecycle with two multicellular stages: cooperative predation and development. During predation, myxobacterial cells produce a wide variety of secondary metabolites and hydrolytic enzymes to kill and consume the prey. It is known that eukaryotic predators, such as ameba and macrophages, introduce copper and other metals into the phagosomes to kill their prey by oxidative stress. However, the role of metals in bacterial predation has not yet been established. In this work, we have addressed the role of copper during predation of *M. xanthus* on *Sinorhizobium meliloti*. The use of biosensors, variable pressure scanning electron microscopy, high-resolution scanning transmission electron microscopy, and energy dispersive X ray analysis has revealed that copper accumulates in the region where predator and prey collide. This accumulation of metal up-regulates the expression of several mechanisms involved in copper detoxification in the predator (the P_1__B_-ATPase CopA, the multicopper oxidase CuoA and the tripartite pump Cus2), and the production by the prey of copper-inducible melanin, which is a polymer with the ability to protect cells from oxidative stress. We have identified two genes in *S. meliloti* (encoding a tyrosinase and a multicopper oxidase) that participate in the biosynthesis of melanin. Analysis of prey survivability in the co-culture of *M. xanthus* and a mutant of *S. meliloti* in which the two genes involved in melanin biosynthesis have been deleted has revealed that this mutant is more sensitive to predation than the wild-type strain. These results indicate that copper plays a role in bacterial predation and that melanin is used by the prey to defend itself from the predator. Taking into consideration that *S. meliloti* is a nitrogen-fixing bacterium in symbiosis with legumes that coexists in soils with *M. xanthus* and that copper is a common metal found in this habitat as a consequence of several human activities, these results provide clear evidence that the accumulation of this metal in the soil may influence the microbial ecosystems by affecting bacterial predatory activities.

## Introduction

*Myxococcus xanthus* is a soil bacterium with a peculiar multicellular behavior, which is observed in all stages of its lifecycle. In the absence of nutrients, cells organize gliding movements to build macroscopic fruiting bodies, with three subpopulations of cells showing division of labor (Muñoz-Dorado et al., 2016). In their natural habitats, they form communities named swarms that feed by preying on a large variety of microorganisms, including Gram-positive and Gram-negative bacteria, and some eukaryotic microorganisms (Pérez et al., 2016). *M. xanthus* swarms move toward their prey and once they are nearby they carry out a cooperative predation process where the bacterial community secretes hydrolytic enzymes and a large number of secondary metabolites that immobilize, kill and degrade the prey. Many of the predatory molecules are packaged within outer membrane vesicles, most likely to facilitate their transport and to reduce the risk of exploitation by cheaters (Whitworth, 2011; Berleman et al., 2014; Remis et al., 2014). The hydrolyzed products are used by the predator to grow (Keane and Berleman, 2016; Muñoz-Dorado et al., 2016; Pérez et al., 2016). Consequently, bacterial predation is a multifaceted process, which depends on numerous parameters associated with both the predator and prey. Although all these elements influence the predatory interaction, most likely none of them is essential for it.

Bacterial predation is increasingly being postulated as a crucial factor in biodiversity due to its potential role in controlling and shaping bacterial populations in the environment (Chase et al., 2002; Gallet et al., 2007; Nair et al., 2019). In bacterial predatory processes, it is not only the predator that plays an active role in attacking and killing the prey, the characteristics of the prey are also decisive. During the progression of the interaction, the predator also prompts a variety of prey responses and adaptations that help to resist or escape predation. *M. xanthus* efficiently preys on several soil bacteria of high agricultural and biotechnological value, such as *Streptomyces*, *Sinorhizobium, Escherichia*, and *Bacillus*. In the case of *Streptomyces coelicolor*, cells respond with antibiotic overproduction and by altering multicellular development (Pérez et al., 2011). The presence of galactoglucan protects *Sinorhizobium meliloti* from being killed by *M. xanthus* (Pérez et al., 2014), and co-evolution experiments with *M. xanthus* and *Escherichia coli* as its prey have demonstrated that the predator induces changes to prey traits such as the production of mucus and the outer membrane protease OmpT (Nair et al., 2019). In the case of *Bacillus*, *B. subtilis* produces bacillaene and spore-filled megastructures in response to predation (Müller et al., 2014, 2015), while *B. licheniformis* escapes from *M. xanthus* predation by deactivating the antibiotic myxovirescin (Wang et al., 2019). A transcriptomic study using *E. coli* as the prey reported that the presence of the predator induced significant changes in 40% of the prey genes. Moreover, five metabolic pathways were significantly up-regulated in *E. coli* upon exposure to outer membrane vesicles, supernatant and/or predatory cells (Livingstone et al., 2018).

These interspecies interactions are also affected by environmental changes or fluctuating concentrations of elements such as metals. Copper is a transition metal that accumulates in soils due to anthropogenic activities, since it is widely used in various industries (electric cables, motors, generators, armaments, water transport, decoration, coins, etc.). On the other hand, the use of copper is widespread due to its broad spectra activity against bacteria, viruses, yeasts and other fungi. It is added as a food supplement to stimulate animal growth by influencing the intestinal microbiota. Copper is used on hospital surfaces since it provides protection against infectious microbes. Currently, copper is also allowed in arboriculture, viticulture, horticulture, extensive agriculture, and even organic agriculture, as a fungicide and herbicide. The European Food Safety Authority (EFSA) has published a report that warns of the high risk posed by copper to birds, mammals and soil organisms. In addition, in recent years it has been proven that non-antibiotic compounds, such as copper, also promote antibiotic resistance through co-selection in terrestrial environments contaminated with this metal (Li et al., 2017; Pal et al., 2017).

Copper is crucial for biological processes and is necessary for the survival of most living organisms. It functions as a cofactor of enzymes that are involved in electron transfer, oxygen transport and redox reactions, and participates in important processes such as respiration, photosynthesis, iron homeostasis, defense against oxidative stress, and pigmentation (Rensing and McDevitt, 2013; Ladomersky and Petris, 2015). This essentiality of copper is due to its ability to oscillate between two oxidation states: Cu(I) and Cu(II). But this characteristic also makes copper toxic to cells. Copper, directly or indirectly, generates oxygen radicals that are responsible for lipid peroxidation, protein oxidation, damage to nucleic acids, and destabilization of iron-sulfur groups in many proteins (Macomber and Imlay, 2009; Waldron et al., 2009; Dupont et al., 2011). This dual effect of copper means that bacteria must regulate the intracellular levels of this metal to meet their physiological needs and avoid damage. This need for cellular control is also exploited by the organisms. For example, metal intoxication (especially with copper) is an antimicrobial strategy used by macrophages in the immune system to reduce the intracellular survival of pathogens (Hodgkinson and Petris, 2012; Djoko et al., 2015; Chandrangsu et al., 2017). In a similar manner, copper is used in the phagosomes of some predator ameba to kill bacteria, contributing to predation. These eukaryotic organisms accumulate copper during phagocytosis by up-regulating genes encoding proteins implicated in copper handling and trafficking, which increases copper levels in the phagosome (Hao et al., 2016). Simultaneously, bacteria respond to this attack by up-regulating the expression of genes involved in copper detoxification (Wolschendorf et al., 2011; Djoko et al., 2015). This interaction indicates that copper response mechanisms in bacteria play crucial roles in pathogenesis and resistance to predation. However, although the use of copper by macrophages and eukaryotic predators to kill the prey and the response of the prey to elude the damage by this metal have been clearly established, the role of copper in bacterial predation remains to be elucidated.

It has recently been reported that the bacterial lifestyle shapes their copper-related proteome, and this is particularly observed in systems involved in copper homeostasis (Antoine et al., 2019). The soil bacterium *Cupriavidus necator*, which is a predator extraordinarily resistant to copper, uses a toxic copper-binding peptide to kill the actinomycete *Agromyces ramosus* (Casida, 1987, 1988), and its predatory activity against *B. subtilis* increases after exposure to copper in a concentration-dependent manner (Seccareccia et al., 2016). *M. xanthus* is the soil bacterium with the most complex copper response known to date (Pérez et al., 2018). Its genome codes for a large number and wide variety of regulatory and structural elements involved in efflux, complexation and oxidation of copper (Sánchez-Sutil et al., 2007, 2013, 2016; Moraleda-Muñoz et al., 2010a, b, 2019; Gómez-Santos et al., 2011; Marcos-Torres et al., 2016; Pérez et al., 2018). Although it has been reported that some of these copper-detoxification systems contribute to completing the developmental cycle of this bacterium upon starvation (Sánchez-Sutil et al., 2007, 2013; Marcos-Torres et al., 2016), it is not clear why this bacterium encodes such a high number of genes to respond to fluctuations in copper concentrations. One possibility could be that *M. xanthus* needs this high abundance of mechanisms related to copper to survive predation by protozoa in soils. However, it is also reasonable to think that *M. xanthus* may utilize copper as part of its arsenal to poison and kill bacterial prey, in a similar manner to eukaryotic predators and macrophages. In this study, we explore this possibility by using *S. meliloti*, an agriculturally important nitrogen-fixing bacterium that contributes to the fertility of soils, as a prey. Our results reveal that copper accumulates in the predatory crossing point, where the two bacteria come into contact, up-regulating the copper detoxification mechanisms in the predator, and triggering the overproduction of melanin in the prey to defend it from predation. These results indicate that copper also plays a role in bacterial predation.

## Materials and Methods

### Bacterial Strains, Plasmids, and Growth Conditions

Genotypes of the *E. coli*, *M. xanthus*, and *S. meliloti* strains, plasmids, and oligonucleotides used in this study are listed in Supplementary Tables S1–S3, respectively. *E. coli* strains were grown in lysogenic broth (Sambrook and Russell, 2001) at 37°C, supplemented with kanamycin (25 μg/ml) when necessary. *M. xanthus* strains were grown in CTT broth (Hagen et al., 1978) at 30°C with vigorous shaking (300 rpm). When needed, X-gal (5-bromo-4-chloro-3-indolyl-β-D-galactopyranoside, 100 μg/ml), kanamycin (80 μg/ml), and/or different metals [CuSO_4_⋅7H_2_O, Cd(NO_3_)_2_⋅4H_2_O, or Zn(NO_3_)_2_⋅6H_2_O] were added to the medium at the concentrations indicated in each figure. *S. meliloti* strains were grown at 30°C in CTT broth, TY complex medium (Beringer, 1974) or defined minimal medium MM (Robertsen et al., 1981), supplemented with kanamycin (200 μg/ml), chloramphenicol (25 μg/ml), sucrose (10%), and/or the concentration of metals [CuSO_4_⋅7H_2_O, Cd(NO_3_)_2_⋅4H_2_O, or Zn(NO_3_)_2_⋅6H_2_O] indicated in each figure, when necessary. Several other bacteria were used to examine copper accumulation in the predatory interface with *M. xanthus*. All of them were grown in lysogenic broth. These bacteria were obtained from the ‘Colección del Departamento de Microbiología’ (Universidad de Granada, Spain) (*Bacillus laterosporus*, *B. subtilis*, *E. coli*, *Micrococcus* sp., *Salmonella* sp., *Staphylococcus aureus*, and *Proteus* sp.), For solid media, Bacto-Agar (Difco) was added at a concentration of 1.5%.

### Nucleic Acid Manipulations

For nucleic acid manipulations, routine molecular biology techniques were used (Sambrook and Russell, 2001). Plasmids were introduced into *E. coli* by transformation (Sambrook and Russell, 2001), into *M. xanthus* by electroporation (Kashefi and Hartzell, 1995), and into *S. meliloti* by conjugation (Simon et al., 1983).

### Predation Experiments

For these experiments, all *M. xanthus* and *S. meliloti* strains were grown in CTT broth and TY broth (with an appropriate antibiotic, when necessary), respectively, with vigorous shaking at 30°C to an optical density at 600 nm (OD_600_) of 1. Cells were centrifuged and concentrated in TM buffer (10 mM Tris-HCl [pH 7.6], 1 mM MgSO_4_) to a final OD_600_ of 15 for *M. xanthus* and 5 for *S. meliloti* strains. Drops of 10 μl of the *S. meliloti* suspensions were deposited onto the surface of CTT agar plates and allowed to dry. Next, drops of 10 μl of the various *M. xanthus* suspensions were spotted in close proximity to the *S. meliloti* spot (no more than 1 mm separation between spots). Plates from 9 replicates for each condition were incubated at 30°C and images from representative samples were taken with a digital camera and an Olympus SZX7 dissecting microscope.

For cell counting and determining the viability of the prey, *M. xanthus* and *S. meliloti* strains were grown as indicated above. However, for this purpose rhizobial cultures were diluted to a final OD_600_ of 0.2. Drops of 10 μl were deposited onto membrane filters (Isopore, Millipore) placed on the surface of CTT agar plates and incubated at 30°C for 24 h. At this time, some filters were collected as described below to obtain the number of cells at 0 h. In the rest of the plates, 10 μl of concentrated culture at an OD_600_ of 15 of the *M. xanthus* DK1622 wild-type strain were deposited on top of the rhizobial colonies. After 24 h of incubation, the filters were deposited in an eppendorf tube, trapped with tube stoppers and thoroughly washed with 1 ml of TM buffer. After centrifugation of the filters for 3 min at 12000 rpm, the supernatants and filters were discarded, and the pellets resuspended in 500 μl of TM buffer. Viable *S. meliloti* cells were counted by using the dilution method onto TY agar supplemented with chloramphenicol, which allows growth of *S. meliloti* strains while inhibiting *M. xanthus* growth. The results are the average of three independent biological replicates. Statistical comparisons between strains were performed using Student’s *t-*test. Differences were considered significant at a level of *p* < 0.05.

For melanin determinations, we used the same procedure mentioned above, except that cells were not spotted on top of a filter. After incubation, cells were directly scraped from the culture media.

### Construction of Strains Harboring *lacZ* Fusions and β-Galactosidase Assays

*Myxococcus xanthus* DK1622 strain was electroporated with plasmids harboring transcriptional fusions between the genes of interest of this myxobacterium (*cuoA*, *copA*, *cus2*, and *czc3*) and *lacZ* from *E. coli* (Supplementary Table S2) to generate the kanamycin-resistant transformants (Supplementary Table S1), which were confirmed by Southern blot analysis. For qualitative β-galactosidase activity analyses during predation, 10-μl drops of *M. xanthus* and *S. meliloti* suspensions at an OD_600_ of 15 and 5 respectively were obtained as indicated above and spotted in close proximity onto CTT agar plates supplemented with X-gal and the metals indicated in the figures. Plates from 9 replicates for each condition were incubated at 30°C. Blue color development as a result of β-galactosidase activity in representative samples was observed with an SZX7 Olympus dissecting microscope.

### Construction of *S. meliloti* In-Frame Deletion Mutants

To generate the *S. meliloti* GR4 *mepA* and *mcoA* deletion mutants, ORF flanking sequences (736-bp upstream and 855-bp downstream of *mepA*, and 782-bp upstream and 799-bp downstream of *mcoA*) were PCR amplified by Phusion High-Fidelity DNA Polymerase (Thermo Fisher Scientific) using *S. meliloti* GR4 genomic DNA as a template and the primers listed in Supplementary Table S3. For the double deletion mutant, sequences 782-bp upstream and 832-bp downstream of the *mcoA* gene were PCR amplified using *S. meliloti* mutant Δ*mepA* genomic DNA as a template and the appropriate primers (Supplementary Table S3). Paired PCR fragments, carrying an internal *Bam*HI (or *Xma*I for the double mutant) and an external *Eco*RI/*Xba*I restriction site, were inserted into the vector pK18*mobsacB*, yielding the plasmids pK18Δ*mepA*, pK18ΔGR4pB023 and pK18ΔGR4pB023.2 (Supplementary Table S2), which were mobilized into the wild-type GR4 strain (or Δ*mepA)* from *E. coli* by conjugation.

Transconjugants arising from a single cross-over event were selected as kanamycin-resistant colonies in MM medium (and simultaneously verified for sucrose sensitivity in TY agar supplemented with 10% sucrose). Kanamycin-resistant/sucrose-sensitive colonies were streaked on sucrose-supplemented TY agar plates to select double cross-over events. The deletion of genes in the colonies thus obtained was checked by PCR using the primers mepA_Efor/mepA_Xrev and SmMCOEfor/SmMCOXrev (or XSmMCOrev2) (Supplementary Table S3), followed by *Bam*HI (or *Xma*I) restriction of the PCR products and sequencing.

### UV-Visible (UV-V) Spectrophotometric Analysis of Extracts Containing Melanin-Type Pigments

Pigments from *S. meliloti* grown as pure cultures or in co-culture with *M. xanthus* (as indicated above for cell counting and viability determinations) on CTT agar plates in the presence of 50 μM copper were extracted following a modified version of the protocol described by Whittaker (1963). Briefly, cells were pelleted in an eppendorf tube and solubilized in 1 ml of 1 N NaOH and 10% dimethyl sulfoxide for 2 h at 80°C. Next, samples were centrifuged at 12000 *g* for 10 min at room temperature, and supernatants were transferred to fresh tubes. Samples were then scanned in a UV-V spectrophotometer (Cary 50 Conc, Varian) at UV and visible wavelengths (230–600 nm). The blank control was 1 N NaOH solution.

### Purification and Fourier Transform Infrared (FT-IR) Spectroscopy of the Melanin-Type Pigment

For these analyses, pigments were purified using the methods described by Sajjan et al. (2010) and Drewnowska et al. (2015). Briefly, solutions containing the pigments extracted as indicated in the previous section were acidified by lowering the pH to 2.0 using 1 N HCl and incubated at room temperature for 1 week, followed by boiling of the suspension for 1 h. Precipitates were then collected by centrifugation at 14000 *g* for 15 min, and pellets were washed three times with 0.1 N HCl and once with double-distilled water. Next, pellets were resuspended in 10 ml ethanol and suspensions were boiled for 10 min and incubated at room temperature for 1 day. Then, samples were centrifuged as above and pellets were washed twice with ethanol. Finally, purified pigments were allowed to dry at room temperature and stored at −20°C until further use.

The FT-IR spectra of the purified pigments were recorded by a JASCO 6200 infrared spectrophotometer. Samples were placed on a diamond window of the spectrometer, and measurements were done in reflection mode, at room temperature, by averaging 32 scans with a resolution of 2 cm^–1^ in the spectra range 400–4000 cm^–1^.

### Microscopy Studies

#### Variable Pressure Scanning Electron Microscopy (VPSEM)

For scanning electron microscopy (SEM), cells from 24-h co-cultures of *M. xanthus* and *S. meliloti* on CTT agar plates were fixed with glutaraldehyde vapors for 24 h at room temperature. Then, samples were washed three times (5 min each) in 0.1 M cacodylate buffer. Dehydration was accomplished by a graded series of ethanol. Samples were then critical-point dried and sputter-coated with gold.

The interface between *M. xanthus* and *S. meliloti* was analyzed by energy dispersive X ray (EDX), imaging secondary electrons (SE) and backscattered electrons (BSE) in a FESEM Zeiss Supra 40Vp equipped with an Aztec 2.1 XMax system. Microanalyses were carried out at 20 kV.

#### High-Resolution Transmission Electron Microscopy (HRTEM)

Samples, taken from the crossing point and from the distal edges of *M. xanthus* and *S. meliloti*, were fixed as described above, and then postfixed with osmium tetroxide. Images and qualitative analysis were obtained in a high-resolution transmission electron microscope FEI TITAN G2 at an acceleration voltage of 300 kV, with a spherical aberration corrector and high-angle annular dark field (HAADF) type detector, which produces an annular dark field image. Resolution in transmission mode was 0.8 Å and in scanning transmission electron microscopy (STEM) 1.3 Å. The microscope is equipped with an XFEG type cannon and a SuperX EDX microanalysis system.

## Results and Discussion

### Copper Helps *M. xanthus* to Penetrate the *S. meliloti* Colony and Induces the Production of a Brown Pigment in the Prey

**FIGURE 1:**
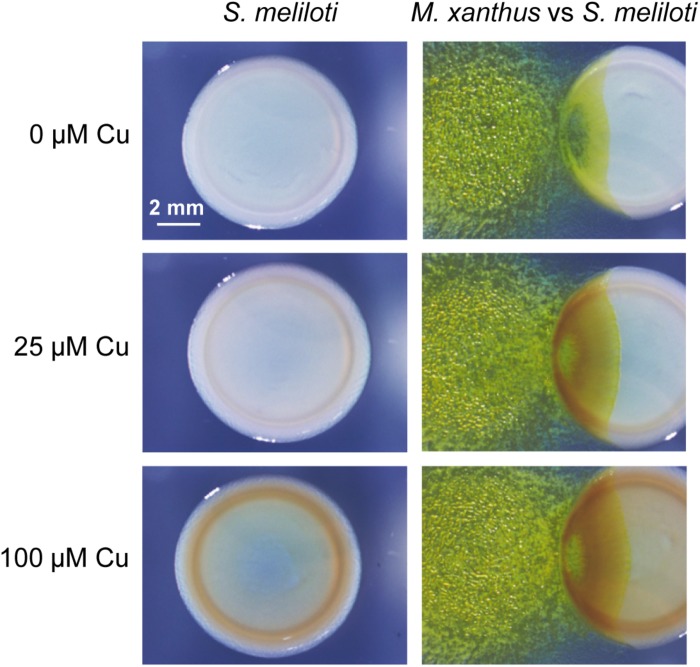
Predatory activity of *M. xanthus* DK1622 versus *S. meliloti* Rm1021 in the presence of different copper concentrations and in the absence of this metal. Pictures were taken at 72 h under a dissecting microscope with illumination from the top of the colonies.

To investigate the role of copper in *M. xanthus* predation, the non-mucoid wild-type strain Rm1021 of *S. meliloti* was tested as a prey by spotting drops of predator and prey next to each other on solid media (see section “Materials and Methods”). Two copper concentrations that are present even in non-contaminated soils were assayed (Sánchez-Sutil et al., 2007). As observed in Figure 1, *M. xanthus* advanced further through the prey colonies as the copper concentration increased, indicating that this metal facilitates the penetration of the predator. Moreover, it was also observed that the yellow color of the *M. xanthus* colonies remained nearly unaltered by the copper concentrations used in these assays. In contrast, a brown color was observed in the *S. meliloti* strain when cells were incubated in media with copper, which was darker at higher concentrations of the metal in both the pure culture and co-culture of prey and predator (Figure 1). However, this color was not observed in media without copper. Furthermore, the brown color was much darker in the area where prey and predator coexist than in the area were the prey has not yet been reached by the predator. Although it might be thought that this color could originate from the superposition of both bacteria, this does not seem to be the case, since the color remains yellow in this region when no copper is added to the medium (Figure 1). These results indicate that copper facilitates *M. xanthus* predation and induces the overproduction of a pigment in the prey, most likely to defend it from the predator attack. Since this pigment is produced in higher amounts in the presence of copper (see panels in Figure 1 with only rhizobia), this observation suggests that this metal accumulates in the region where predator and prey overlap. An explanation of why *M. xanthus* penetrates the prey colonies more easily in the presence of copper could be that these copper concentrations stimulate growth. However, it has been reported that growth rates of *M. xanthus* remains nearly constant with copper concentrations between 0 and 500 μM copper (Sánchez-Sutil et al., 2007; Gómez-Santos et al., 2012). In contrast, adventurous and social motility in this myxobacterium are stimulated by the copper concentrations used in this study (Gómez-Santos et al., 2012). Taking into consideration that both types of motility are required for proper predation, since mutants in any motility system prey with three order of magnitude less efficiently than the wild-type strain on *S. meliloti* Rm1021 (Pérez et al., 2014), stimulation of A and S motility by copper might facilitate penetration of *M. xanthus* on the prey colony.

### Copper Accumulates in the Predatory Crossing Point

**FIGURE 2:**
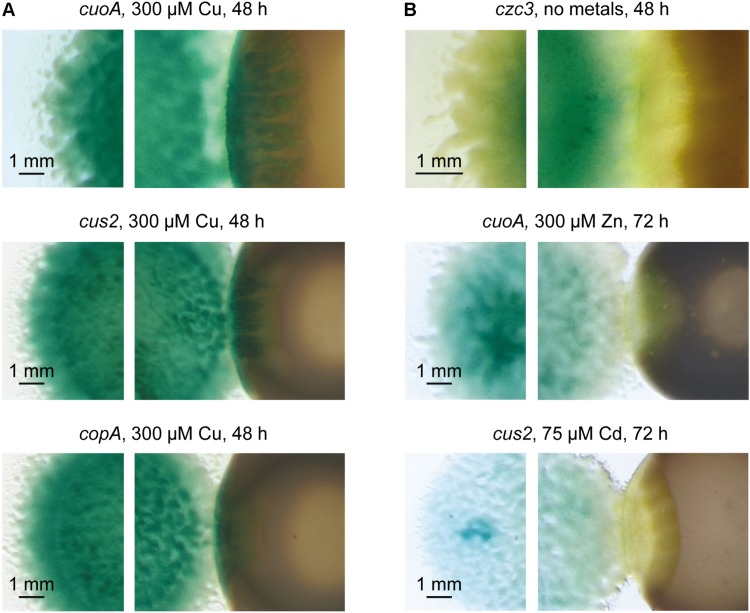
Detection of copper accumulation in the predatory interface of *M. xanthus* and *S. meliloti* by using *M. xanthus* copper biosensors strains. **(A)** *M. xanthus* strains harboring fusions between genes *cuoA*, *cus2* and *copA*, and *lacZ* were co-cultured with *S. meliloti* Rm1021 in the presence of copper. **(B)** As negative controls, *M. xanthus* strains harboring fusions between genes *czc3*, *cuoA*, and *cus2*, and *lacZ* were co-cultured with *S. meliloti* Rm1021 in the absence of metals, zinc, and cadmium, respectively. Strains were spotted onto CTT agar plates containing 100 μg/ml X-gal to visualize the blue color development. Pictures were taken after 48 or 72 h of incubation under a dissecting microscope with illumination from the bottom of the colonies.

Several types of analyses were carried out to find out whether copper accumulates in the region where predator and prey collide. First, several strains harboring fusions between promoters of metal-responsive and non-responsive genes and *lacZ* were constructed in *M. xanthus* DK1622 to be used as biosensors (Supplementary Table S1). The genes chosen for these analyses were *cuoA*, *copA*, *cus2*, and *czc3* (Sánchez-Sutil et al., 2007; Moraleda-Muñoz et al., 2010a, b). *cuoA* and *copA* encode a multicopper oxidase (MCO) and a P_1__B_-type ATPase respectively, whose expressions have been reported to be dependent on copper and other divalent metals, such as zinc (Sánchez-Sutil et al., 2007; Moraleda-Muñoz et al., 2010b). In contrast, the systems Cus2 and Czc3 are both tripartite complexes involved in heavy metal efflux. However, while the expression of genes of the Cus2 system is dependent on copper and cadmium, genes of the Czc3 system exhibit a constitutive metal-independent expression. The fusion constructed in the Czc3 system was used as a negative control (Moraleda-Muñoz et al., 2010a). The biosensor strains and *S. meliloti* Rm1021 were then spotted onto CTT agar plates containing X-gal and different copper and other metal concentrations to visualize the blue color development in different regions of the co-culture. As shown in the photographs on the left of Figure 2A, cells harboring fusions with the genes *cuoA*, *copA* and *cus2* incubated in the presence of copper that glide from the original spot to a region where the prey is not present develop a lighter blue color, probably as a result of lower density and shorter time to hydrolyze X-gal. In contrast, when they approach and enter the prey colony, the blue color is much darker, and similar to that observed in the center of the original spot (Figure 2A, right photographs). It could be argued that the cell density of the predator in the predator-prey interface is higher than in other surrounding regions due to the consumption of the prey. However, the intensity of the blue color is quite similar in all the regions around the original spot in the case of the *M. xanthus* strain harboring the fusion in the constitutive *czc3* system (compare photographs on left and right of Figure 2B, upper part), indicating that this does not seem to be the case. Moreover, blue color accumulation was not detected at the crossing zones when strains harboring fusions in the *cuoA* and *cus2* genes were incubated in the presence of zinc and cadmium respectively (Figure 2B, compare photographs on left and right where these two strains are shown), in spite of the fact that these genes are also up-regulated by these metals (Sánchez-Sutil et al., 2007; Moraleda-Muñoz et al., 2010a). These observations seem to indicate that copper accumulates during predation where *M. xanthus* makes contact with *S. meliloti*. Copper accumulation was also observed in the predatory interface of *M. xanthus* with other Gram-negative (*Proteus*, *Salmonella*, and *E. coli*) and Gram-positive (*Micrococcus*) bacteria. However, during the interaction of *M. xanthus* with other Gram-positive bacteria blue color accumulation was not apparent (*B. laterosporus* and *S. aureus*) or was absent (*B. subtilis*) (Supplementary Figure S2), indicating that copper accumulates only in some interactions predator-prey.

**FIGURE 3:**
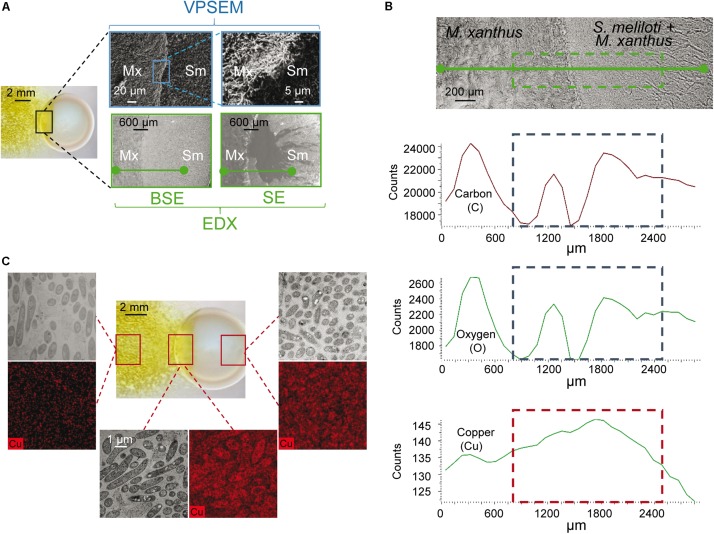
Determination of copper by electron microscopy at the interaction crossing point of *M. xanthus* and *S. meliloti* in the presence of 10 μM copper. **(A)** Pictures of the region where predator and prey collide were taken with a dissecting microscope (left picture), a variable pressure scanning electron microscope (VPSEM), and a VPSEM equipped with an electron dispersive X ray (EDX). Pictures for EDX were taken by backscattered electrons (BSE), which carries information about the composition of the sample, and secondary electrons (SE), which inspects the topography of the sample surface. Squares indicate the section used to prepare the samples or where the different image amplifications were taken. **(B)** Semi-quantification of carbon, oxygen and copper by using VPSEM in the selected section (green segment) represented in the upper photograph taken with EDX. **(C)** High resolution transmission microscopy with EDX microanalysis for copper (black and red pictures) in the regions shown with squares in the image taken under a dissecting microscope (central picture). Black and white images of the same area were taken with a high resolution scanning transmission electron microscope using a HAADF detector. Samples for microscopy preparations were taken at 48 h.

A second method for determining whether copper accumulates in the region where predator and prey are in contact was SEM coupled with EDX. This method was applied in the area of contact (Figure 3A, delineated in blue), and a segment (drawn in green in Figures 3A,B) was chosen for the scan microanalysis of different chemical elements. Carbon and oxygen were analyzed to determine the quantity of cells in this area. As can be observed in Figure 3B, these two elements exhibit non-linear profiles, as expected from the stacking of bacteria when the two fronts collide, which is observed in Figure 3A. However, copper exhibited a different profile, since the amount of this metal increases as *M. xanthus* approaches and makes contact with the prey, then decreases at greater distances, where more prey and less predator are present. Finally, to further investigate whether copper accumulates at the crossing point, samples from the distal edges of the predator and prey, and from the interface zone, were prepared and examined by using HRTEM coupled to an EDX microanalysis system. As observed in Figure 3C, the copper signal (images in red) was of higher intensity at the crossing point than at the distal edges, where only prey or predator are present. These results corroborate the accumulation of copper at the contact point as a consequence of interaction.

The results obtained with copper biosensors and EDX microanalyses indicate that copper accumulates in the region where the predator and prey make contact, and suggest that there must be an alteration of copper trafficking in the predatory intersection zone.

### *S. meliloti* Overproduces Melanin in Response to the Invasion by *M. xanthus*

As mentioned above, a brown pigment is produced by *S. meliloti* Rm1021 in the presence of copper, exhibiting a higher intensity in the region where the prey and predator coexist (Figure 1). We had several reasons to suspect that this pigment might be the aromatic polymer melanin. (1) The production of a melanin-like pigment has been previously reported in several rhizobia (Cubo et al., 1988), including *S. meliloti* GR4, whose expression is dependent on copper (Mercado-Blanco et al., 1993; Castro-Sowinski et al., 2002). (2) An important biological role invoked for melanins is metal chelation (Hong and Simon, 2007), including copper, which is sequestered by this polymer to protect cells from oxidative stress (Korytowski et al., 1985; Hamilton and Gomez, 2002). (3) Melanins are also involved in resistance to attack by different cell-wall enzymes (Kuo and Alexander, 1967; Rosas and Casadevall, 2001; Steenbergen et al., 2001), improving survival and competitive skills under several environmental stresses (Bell and Wheeler, 1986; Coyne and Al-Harthi, 1992; Nosanchuk and Casadevall, 2003; Zhang et al., 2007). These previous studies prompted us to determine whether the brown pigment produced by *S. meliloti* in the presence of copper, and especially at the predator-prey interface, could be melanin, which would function as a defensive mechanism against predation.

Mercado-Blanco et al. (1993) reported that *S. meliloti* GR4 produced melanin in the presence of copper and tyrosine, and demonstrated that when the gene for the tyrosinase MepA, encoded in the plasmid pRmeGR4b, was expressed in *Agrobacterium tumefaciens* and *E. coli*, these bacteria produced melanin. Since no studies about melanin biosynthesis are available for the strain Rm1021, to determine whether the brown pigment observed in the co-culture of *M. xanthus* and *S. meliloti* in the presence of copper was this complex and varied phenolic polymer, we decided to use the GR4 strain of *S. meliloti* instead of Rm1021 as the prey. First, we checked whether the strain GR4 behaved like the strain Rm1021. The results obtained revealed that *M. xanthus* penetrated more easily in the colony of this rhizobial strain, that the GR4 strain produced a brown pigment in the presence of copper, with highest intensity at the prey-predator interface, and that the biosensor strains of *M. xanthus* harboring fusions between the copper-inducible genes *copA*, *cuoA* and *cus2*, and *lacZ* also exhibited the highest β-galactosidase activity in the area where they collide with *S. meliloti* GR4 (Supplementary Figure S1).

**FIGURE 4:**
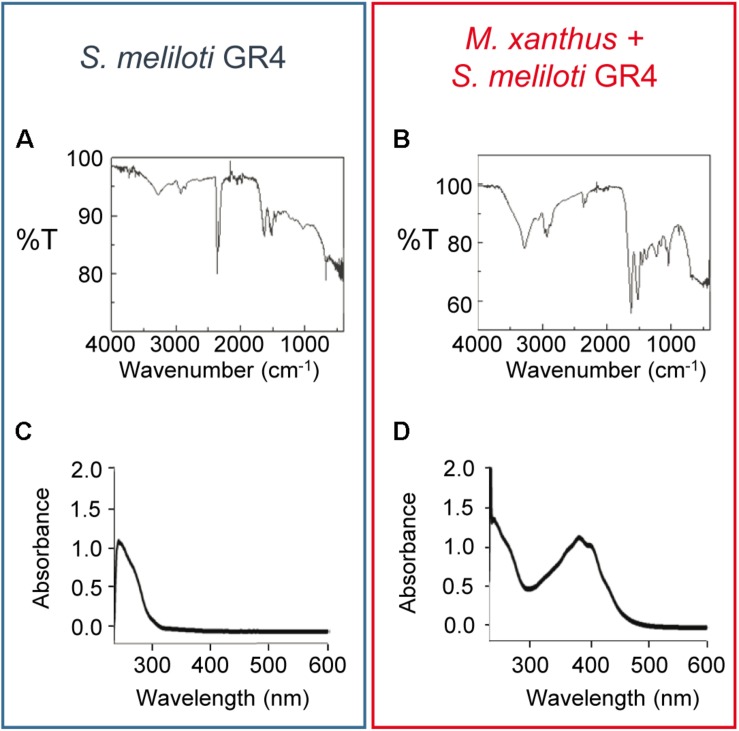
Analysis of the brown pigment produced by *S. meliloti* GR4 in the presence of copper. FT-IR **(A,B)** and UV-V **(C,D)** spectra of the dry pigment extracted from pure cultures of *S. meliloti* GR4 (blue rectangle) and co-cultures with *M. xanthus* (red rectangle). Extracts were obtained after 48 h of incubation on CTT agar plates supplemented with 50 μM copper.

Next, pigment was extracted from *S. meliloti* GR4 grown on pure culture and co-culture with *M. xanthus* in the presence of copper after 48 h of incubation. Extracts were then analyzed by FT-IR spectroscopy. As shown in Figures 4A,B, the FT-IR spectra obtained of the solid brown pigment extracted in both samples were very similar. Moreover, these spectra exhibited several features that are identical to those described for bacterial melanins of *E. coli* (Chávez-Béjar et al., 2013; Mejia-Caballero et al., 2016), *Lysobacter oligotrophicus* (Kimura et al., 2014), *Azotobacter chroococcum* (Banerjee et al., 2014), *Pseudomonas* (Tarangini and Mishra, 2013), *Streptomyces cyaneofuscatus* (Al Khatib et al., 2018), *Bacillus weihenstephanensis* (Drewnowska et al., 2015), and soil bacteria (Tarangini and Mishra, 2014). Nevertheless, to further confirm that the pigment was melanin, the absorbance in UV-V of pigments extracted in NaOH from a pure culture of *S. meliloti* GR4 and a co-culture with *M. xanthus* following a modified version of the protocol described by Whittaker (1963) (see section “Materials and Methods” for details) was also determined. As shown in Figures 4C,D, the spectra obtained resemble those described for other melanins (Szpoganicz et al., 2002), exhibiting a peak at 243 nm. In the extracts from the co-culture of both bacteria, additional peaks were detected with maxima at 386–405 nm (Figure 4D). Those peaks are most likely due to the *M. xanthus* yellow pigment DKxanthene, which belongs to a family of secondary metabolites that shows maxima of absorption around 400 nm (Meiser et al., 2006).

### Identification of *S. meliloti* GR4 Genes Involved in Melanin Biosynthesis

**FIGURE 5:**
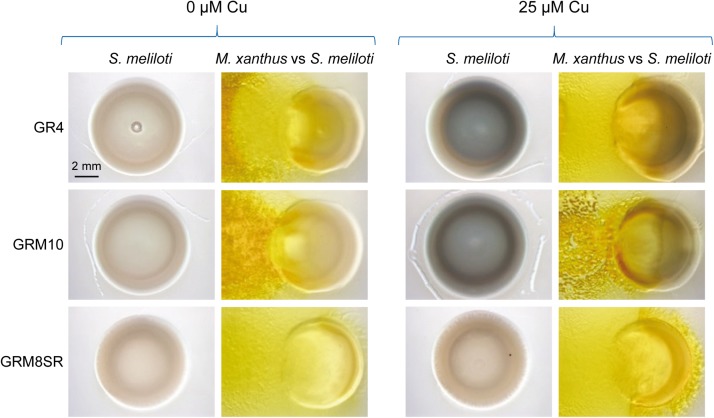
Melanin production by *S. meliloti* GR4 and its derivatives GRM10 and GRM8SR strains in the absence and the presence of copper in pure cultures and co-culture with *M. xanthus*. Cells were incubated for 72 h. Then, 30 μl of SDS at 10% was added to the colonies, and plates were incubated for 24 h at room temperature prior to taking photographs under a dissecting microscope with illumination from the top of the colonies.

*Sinorhizobium meliloti* GR4 (Casadesús and Olivares, 1979) holds a genome with a total length of 7.15 Mb (Martínez-Abarca et al., 2013), comprising the chromosome (3.62 Mb), two small stable plasmids, pRmeGR4a (0.18 Mb) and pRmeGR4b (0.23 Mb), and two pSym plasmids, pRmeGR4c (1.42 Mb) and pRmeGR4d (1.70 Mb). As mentioned above, Mercado-Blanco et al. (1993) demonstrated that *mepA* encodes a tyrosinase involved in melanin biosynthesis, which is included in plasmid pRmeGR4b. However, taking into consideration that in their experiments they used 10 times as much copper as we did (25 μM copper versus 250 μM), that they needed to supplement the culture media with tyrosine (which was not included in our assays), and that they used pure cultures while we observed the highest intensity of brown color in the co-culture of *S. meliloti* with *M. xanthus*, we decided to carry out a number of experiments to identify the genes that are involved in melanin biosynthesis when the rhizobium has to defend itself from the predator. First, *M. xanthus* was assayed against the strains of *S. meliloti* GRM8SR and GRM10, both derived from GR4 (Mercado-Blanco et al., 1993). GRM10 possesses the plasmids pRmeGR4b, pRmeGR4c and pRmeGR4d, while GRM8SR only contains the plasmids pRmeGR4c and pRmeGR4d (Supplementary Table S1). As can be observed in Figure 5, the strains GR4 and GRM10 synthesize melanin, and only in the strain GRM8SR is the dark pigment not observed. The same result is observed in pure cultures and co-cultures, indicating that the melanin biosynthetic enzymes that are up-regulated during predation in the presence of copper are encoded in the non-symbiotic plasmid pRmeGR4b, where the gene *mepA* is located (Mercado-Blanco et al., 1993).

**FIGURE 6:**
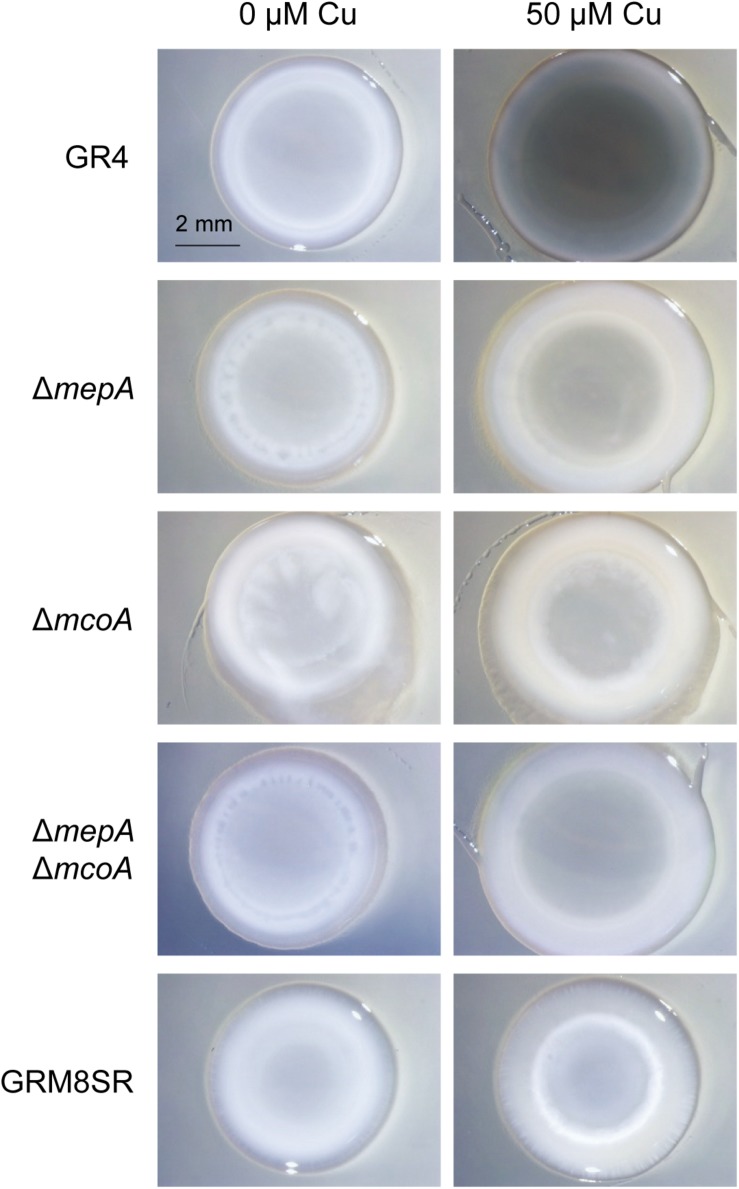
Melanin production in different *S. meliloti* strains. The wild-type strain GR4, the curated strain GRM8SR, and the mutants Δ*mepA*, Δ*mcoA*, and Δ*mepA*Δ*mcoA* were analyzed for melanin production in pure cultures. Cells were spotted onto CTT agar plates containing 0 and 50 μM copper and incubated for 72 h. Then 30 μl of 10% SDS was added on top of the colonies, and they were incubated for 24 h at room temperature. Photographs were taken under a dissecting microscope with illumination from the top of the colonies.

Mercado-Blanco et al. (1993) demonstrated the tyrosinase activity of *mepA* by expressing it in *E. coli* and analyzing the extracts in non-denaturing gels. To study the role of MepA in melanin biosynthesis in *S. meliloti* and in protection against predation, an in-frame deletion Δ*mepA* mutant was constructed (Supplementary Table S1). The analysis of this mutant for melanin production revealed that the amount of SDS-extractable brown pigment was much smaller than in the wild-type strain (Figure 6). However, the color of the mutant was slightly darker than that observed in the GRM8SR strain, indicating that other gene(s) must remain in the pRmeGR4b plasmid with the ability to synthesize melanin. To identify other genes in this plasmid involved in the production of melanin, a bioinformatic analysis of the plasmid sequence was carried out (Supplementary Table S4). Sequences of all open reading frames included in this plasmid (accession number CP003935; Martínez-Abarca et al., 2013) were reanalyzed, searching for domains in the Pfam database (Finn et al., 2015), functions of the proteins and metabolic pathways in the KEGG database (Kanehisa and Goto, 2000), and BLAST analyses in the NCBI database^1^. The most plausible candidate to be involved in melanin biosynthesis was the gene C770_GR4pB023 (designated *mcoA*), which encodes an MCO with the domains Cu-oxidase_3 and Cu-oxidase_2 in the Pfam database, and the four copper-binding domains typical of MCOs (Cha and Cooksey, 1991; Brown et al., 1995; Outten et al., 2000; Sánchez-Sutil et al., 2007). Some MCOs exhibit laccase activity and are able to synthesize melanin in fungi and bacteria (Hullo et al., 2001; Castro-Sowinski et al., 2002, 2007; Martins et al., 2002; Hernández-Romero et al., 2005; Sapmak et al., 2015; Li et al., 2016). Our analyses also revealed that this gene forms a cluster with a hypothetical protein (C770_GR4pB024) and *mepA* (C770_GR4pB025). These three genes conserve microsynteny (according to KEGG database) with other *S. meliloti* strains, such as BL255C, AK83 and SM1. Synteny is also conserved with other *Sinorhizobium* strains and related genera (*S. medicae* and *S. fredii* HH10, *Bradyrhizobium* sp. CCGE-LA001, *Mesorhizobium ciceri*, *M*. *opportunistum* and *M. australicum*), and in other non-related bacteria such as *Caulobacter flavus* and *Granulicella mallensis*. The conservation of the genomic architecture suggests that these three genes are implicated in the same function.

To investigate the role of this MCO in melanin biosynthesis and predation, a Δ*mcoA* mutant was constructed (Supplementary Table S1), and the analysis of the SDS-extractable pigment in the generated mutant revealed that deletion of this gene also diminishes melanin production, as the Δ*mepA* mutant (Figure 6). This observation led us to construct a double mutant Δ*mepA*Δ*mcoA*, and the analysis of this strain indicated that it is non-melanogenic, as the strain GRM8SR. Therefore, it can be concluded that both McoA and MepA are responsible for melanin biosynthesis in *S. meliloti* GR4 (Figure 6).

### Melanin Plays a Defensive Role Against Predation

**FIGURE 7:**
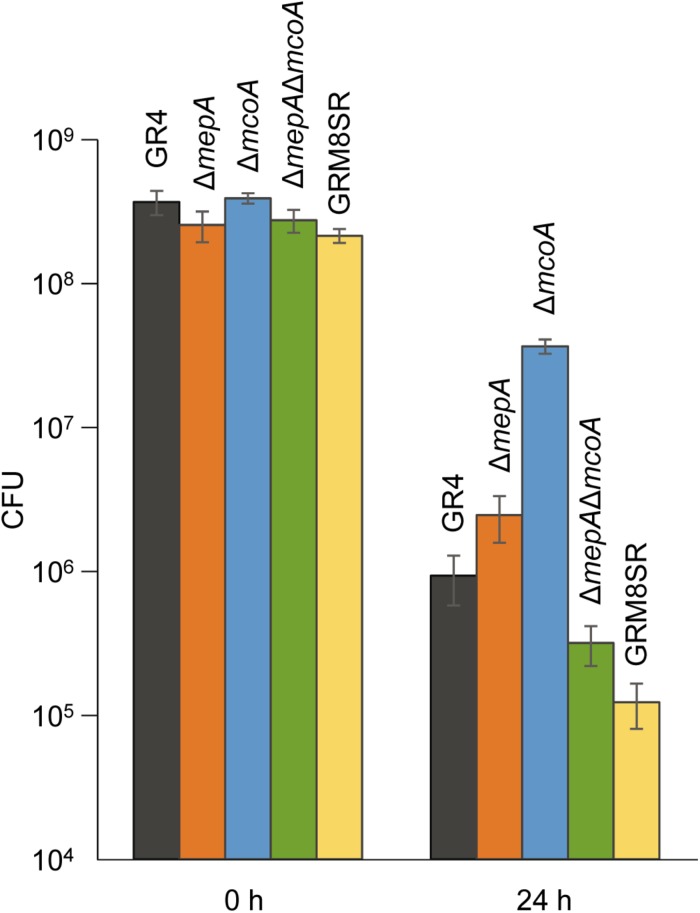
Survivability of the *S. meliloti* strains GR4, GRM8SR, Δ*mepA*, Δ*mcoA*, and Δ*mepA*Δ*mcoA* to predation by *M. xanthus*. Viable cells were counted by using the dilution method, and the number of colony forming units (CFU) obtained at 0 and 24 h of co-culture is shown. Experiments were performed in triplicate. Error bars indicate standard deviations.

To determine the role of *S. meliloti* melanin as a defensive mechanism against predation, the number of cells that survive the predatory activity of *M. xanthus* was determined using the wild-type strain GR4, the curated strain GRM8SR, and the three mutants generated, Δ*mepA*, Δ*mcoA*, and Δ*mepA*Δ*mcoA*, as prey. These quantitative studies were carried out according to the method described by Pérez et al. (2014), in which predator cells were spotted on top of the prey, instead of placing two drops in close proximity to one another. With this technique, predation can start immediately, because predator cells do not have to glide to reach the prey. Moreover, the reproducibility of these assays is higher, because it is not affected by small differences in the distance between prey and predator. As shown in Figure 7, after 24 h of interaction the two single mutants were more resistant to predation (*p*-values using Student’s *t-*test of 0.0469 for Δ*mepA* and 0.0001 for Δ*mcoA*) than the wild-type strain. However, it is known that cells exhibit several types of defensive mechanisms against an excess of copper (Pérez et al., 2018) and that, in some cases, deletion of only one of them yields cells that are more resistant to this metal because one or several of the other mechanisms are overexpressed due to an imbalance in copper trafficking. For instance, in the case of *M. xanthus*, it has been shown that deletion of two P_1__B_-type ATPases (CopA and CopB) yielded cells more resistant to copper than the wild-type strain, in spite of the fact that this type of protein pumps copper from the cytoplasm to the periplasm (Moraleda-Muñoz et al., 2010b). An in-depth study to identify reasons for this increase in resistance to the metal revealed that it originated in a high up-regulation of the *cus2* and *cus3* systems in the mutant, which extrude copper to the exterior in a more efficient manner than ATPases (Moraleda-Muñoz et al., 2010b). This could also be the situation in the strains where *mepA* and *mcoA* are deleted, although genes that can be overexpressed in these mutants have not been identified, since the copper response in *S. meliloti* GR4 has not been addressed. Next, the double mutant Δ*mepA*Δ*mcoA* and the GRM8SR strains were also used to determine their sensitivity to predation. On the other hand, after 24 h of interaction the double mutant and the curated strain of plasmids pRmeGR4a and pRmeGR4b turned out to be more sensitive to the predation (*p*-values of 0.0440 and 0.0169, respectively) than the wild-type strain, indicating that melanin protects the prey from predation (Figure 7). As the GRM8SR strain is more sensitive to predation than the double mutant Δ*mepA*Δ*mcoA*, it is expected that some other genes encoded in the two plasmids that are lacking in this strain also contribute to providing resistance against predation. Although the differences observed in cell viability are not very noticeable, it should be taken into consideration that this rhizobium encodes other genes that are predicted to contribute to copper resistance, and that melanin is only one of several mechanisms used by cells to protect themselves from oxidative stress.

## Conclusion

Bacterial predation is considered a complex microbial interaction that may have implications in the environment, diversity of organisms, and clinical, agricultural and biotechnological applications. However, although much effort has been dedicated to untangling this process, we are still far from understanding all the mechanisms used by the predator to kill and consume the prey, and those used by the prey to protect itself from the predator. The results presented here demonstrate that copper accumulates during predation at the interface where the predator and prey coexist. Because of this accumulation, we have shown that several mechanisms known to be involved in protection against copper, whose expression depends on this metal, are up-regulated in both the prey and predator. Thus, *M. xanthus* up-regulates the expression of the genes for the P_1__B_-ATPase CopA, the MCO CuoA, and the tripartite pump Cus2, all of which are involved in copper detoxification. The accumulation of copper at the point where both bacteria collide is also the cause of the observed melanin overproduction by the prey in this region. Melanins are pigments known to protect against oxidative stress, and in fact we have found here that melanin protects the prey from predation in the presence of copper. Moreover, we have identified that melanin biosynthesis in *S. meliloti* GR4 is carried out by at least two genes, *mepA* and *mcoA*, encoded in the plasmid pRmeGR4b. Although these data have revealed that copper also plays a role in bacterial predation, it remains to be elucidated which bacterium is responsible for the accumulation of this metal, and which mechanisms are used by cells to achieve the deposition of the metal only in the specific region where the prey and predator coexist. Consequently, more experiments will be required to elucidate the exact role of copper in bacterial predation.

## Data Availability Statement

The datasets generated for this study are available on request to the corresponding author.

## Author Contributions

FC-M performed the qualitative and quantitative predation experiments, construction of the strains harboring *lacZ* fusions, β-galactosidase assays, melanin analyses, and microscopy studies designed the experiments, and critically read the manuscript. JM-D designed and supervised the experiments, wrote the manuscript, and performed the experiments for the construction of strains harboring *lacZ* fusions, β-galactosidase assays, and melanin analyses. NG-T performed the predation experiments, designed and constructed the *S. meliloti* mutants, and critically read the manuscript. GM-N constructed the strains harboring *lacZ* fusions and critically read the manuscript. JP wrote the manuscript, designed and performed the quantitative predation experiments, and carried out the microscopy studies. AM-M designed and supervised all the experiments, wrote the manuscript, and performed the qualitative and quantitative predation experiments, construction of the strains harboring *lacZ* fusions, β-galactosidase assays, and melanin analyses.

## Conflict of Interest

The authors declare that the research was conducted in the absence of any commercial or financial relationships that could be construed as a potential conflict of interest.

## Footnotes

1 http://blast.ncbi.nlm.nih.gov/Blast.cgi
