# Supplementary material for: Copper and Melanin Play a Role in Myxococcus xanthus Predation on Sinorhizobium meliloti
Source: Front Microbiol. 2020 Feb 4;11:94. doi: 10.3389/fmicb.2020.00094 (PMC7010606; doi:10.3389/fmicb.2020.00094)
Supplement: Supplementary file 1 [file Data_Sheet_1.docx]

Table S1. Bacterial strains used in this study.

| **Bacterial strains** | **Genotype/phenotype^a^** | **Reference or source** |
| --- | --- | --- |
| *E.coli* |  |  |
| S17.1 | *recA pro hsdR RP4-2-Tc::Mu-Km::Tn7* | Simon et al., 1983 |
| DH5α | F^–^ *endA1* *glnV44* *thi-1* *recA1* *relA1* *gyrA96* *deoR* *nupG* *purB20* φ80d*lacZ*ΔM15 Δ(*lacZYA-argF*)U169, hsdR17(*r_K_*^–^*m_K_*^+^), λ^–^ | Bethesda Research Lab |
|  |  |  |
| *M. xanthus* |  |  |
| DK1622 | Wild type | Kaiser, 1979 |
| JM51AZYDK | *cuoA-lacZ* Km^r^ | This study |
| JMCAlacDK | *copA-lacZ*, Km^r^ | This study |
| JMCus2lacDK | *cus2-lacZ*, Km^r^ | This study |
| JMCzc3lacDK | *czc3-lacZ*, Km^r^ | This study |
|  |  |  |
| *S. meliloti* |  |  |
| Rm1021 | SU47 derivative (reference strain) | Meade et al., 1982 |
| GR4 | Wild type; Chl^r^ | Casadesús and Olivares, 1979 |
| GRM8SR | pRmeGR4a- and pRmeGR4b-cured derivative of GR4; Chl^r^ | Mercado-Blanco et al., 1993 |
| GRM10 | pRmeGR4a-cured derivative of GR4; Chl^r^ | Mercado-Blanco et al., 1993 |
| Δ*mepA* | Δ*mepA*; Km^s^, Chl^r^ | This study |
| Δ*mcoA* | Δ*mcoA*; Km^s^, Chl^r^ | This study |
| Δ*mepA*Δ*mcoA* | Δ*mepA-*Δ*mcoA*; Km^s^, Chl^r^ | This study |

^a^Km^r^ and Km^s^ indicate resistance and sensitivity to kanamycin, respectively. Chl^r^ indicates chloramphenicol resistance.

Table S2. Plasmids used in this study.

| **Plasmid** | **Genotype** | **Reference or source** |
| --- | --- | --- |
| pKY481-CuoA | *cuoA-lacZ,* Km^r^ | Sánchez-Sutil et al., 2007 |
| pAELCopAlac | *copA-laZ*, Km^r^ | Moraleda-Muñoz et al., 2010b |
| pAELCus2lac | *cus2-lacZ*, Km^r^ | Moraleda-Muñoz et al., 2010a |
| pAELCzc3lac | *czc3-lacZ*, Km^r^ | Moraleda-Muñoz et al., 2010a |
| pK18*mobsacB* | Suicide plasmid in *S. meliloti*; *sacB*, *oriV*, Km^r^ | Schäfer et al., 1994 |
| pK18Δ*mepA* | Suicide plasmid for GR4pB025 deletion; Km^r^ | This study |
| pK18Δ*mcoA* | Suicide plasmid for GR4pB023 deletion; Km^r^ | This study |
| pK18Δ*mcoA*.2 | Suicide plasmid for GR4pB023 deletion in *S. meliloti* GR4 Δ*mepA* ; Km^r^ | This study |

Table S3. Oligonucleotides used in this study.

| **Oligonucleotide** | **For amplification of** | **Sequence (5’→3’)^a^** |
| --- | --- | --- |
| mepA_Efor | Upstream of *mepA* | CATCGAATTCTGGTGCCAATGATGACGG |
| mepA_Brev | Upstream of *mepA* | CGATGGATCCGTTCTTGCGCCTATGCTTGG |
| mepA_Bfor | Downstream of *mepA* | GAATGGATCCACCGCGCGAGGCACAGAAAT |
| mepA_Xrev | Downstream of *mepA* | CGGATCTAGAGACCCGGTACACTATGTCACC |
| SmMCOEfor | Upstream of *mco* | GTGGAATTCTACTTCGGCGACCTCATACA |
| SmMCOBrev | Upstream of *mco* | AGAGGATCCCAATCAAACCTCCCAAACGT |
| SmMCOBfor | Downstream of *mco* | GGCGGATCCTAGATAGCTGATCGCCTTTT |
| SmMCOXrev | Downstream of *mco* | GTTTCTAGAAAGTCTCTTCGAAATAGCCA |
| SmMCOXmarev | Upstream of *mco* in GR4 Δ*mepA* | AGACCCGGGCAATCAAACCTCCCAAACGT |
| SmMCOXmafor | Downstream of *mco* in GR4 Δ*mepA* | GGCCCCGGGTAGATAGCTGATCGCCTTTT |
| XSmMCOrev2 | Downstream of *mco* in GR4 Δ*mepA* | CCATCTAGAAACGGACCGGTCACGATATT |

^a^Underlined are the restriction sites used in cloning.

**REFERENCES**

Casadesús, J., and Olivares, J. (1979). Rough and fine linkage mapping of the *Rhizobium meliloti* chromosome. *Mol. Gen. Genet.* 174, 203‐209. doi: 10.1007/bf00268356.

Kaiser, D. (1979). Social gliding is correlated with the presence of pili in *Myxococcus xanthus. Proc.* *Natl. Acad. Sci. U. S. A.* 76, 5952-5956. doi: 10.1073/pnas.76.11.5952.

Meade, H. M., Long, S. R., Ruvkun, G. B., Brown, S. E., and Ausubel, F. M. (1982). Physical and genetic characterization of symbiotic and auxotrophic mutants of *Rhizobium meliloti* induced by transposon Tn5 mutagenesis. *J. Bacteriol.* 149, 114-122.

Mercado-Blanco, J., García, F., Fernández-López, M., and Olivares, J. (1993). Melanin production by *Rhizobium meliloti* GR4 is linked to nonsymbiotic plasmid pRmeGR4b: cloning, sequencing, and expression of the tyrosinase gene *mepA*. *J. Bacteriol.* 175, 5403-5410. doi: 10.1128/jb.175.17.5403-5410.1993.

Moraleda-Muñoz, A., Pérez, J., Extremera, A. L., and Muñoz-Dorado, J. (2010)a. Differential regulation of six heavy metal efflux systems in the response of *Myxococcus xanthus* to copper. *Appl. Environ. Microbiol*. 76, 6069-6076. doi: 10.1128/AEM.00753-10.

Moraleda-Muñoz, A., Pérez, J., Extremera, A. L., and Muñoz-Dorado, J. (2010)b. Expression and physiological role of three *Myxococcus xanthus* copper dependent P_1B_-type ATPases during bacterial growth and development. *Appl. Environ. Microbiol*. 76, 6077-6084. doi: 10.1128/AEM.00755-10.

Sánchez-Sutil, M. C., Goómez-Santos, N., Moraleda-Muñoz, A., Martins, L. O., Pérez, J., and Muñoz-Dorado, J. (2007). Differential expression of the three multicopper oxidases from *Myxococcus xanthus. J. Bacteriol*. 189, 4887-4898. doi: 10.1128/JB.00309-07.

Schäfer, A., Tauch, A., Jager, W., Kalinowski, J., Thierbach, G., and Puhler, A. (1994). Small mobilizable multi-purpose cloning vectors derived from the *Escherichia coli* plasmids pK18 and pK19: selection of defined deletions in the chromosome of *Corynebacterium glutamicum*. *Gene.* 145, 69-73. doi: 10.1016/0378-1119(94)90324-7.

Simon, R., Priefer, U., and Pehle, A. (1983). A broad host range mobilization system for *in vivo* genetic engineering: transposon mutagenesis in gram negative bacteria. *Nat. Biotechnol.* 1, 784-791. doi:10.1038/nbt1183-784.
