# Supplementary material for: Copper and Melanin Play a Role in Myxococcus xanthus Predation on Sinorhizobium meliloti
Source: Front Microbiol. 2020 Feb 4;11:94. doi: 10.3389/fmicb.2020.00094 (PMC7010606; doi:10.3389/fmicb.2020.00094)
Supplement: Supplementary file 3 [file Image_1.PDF]

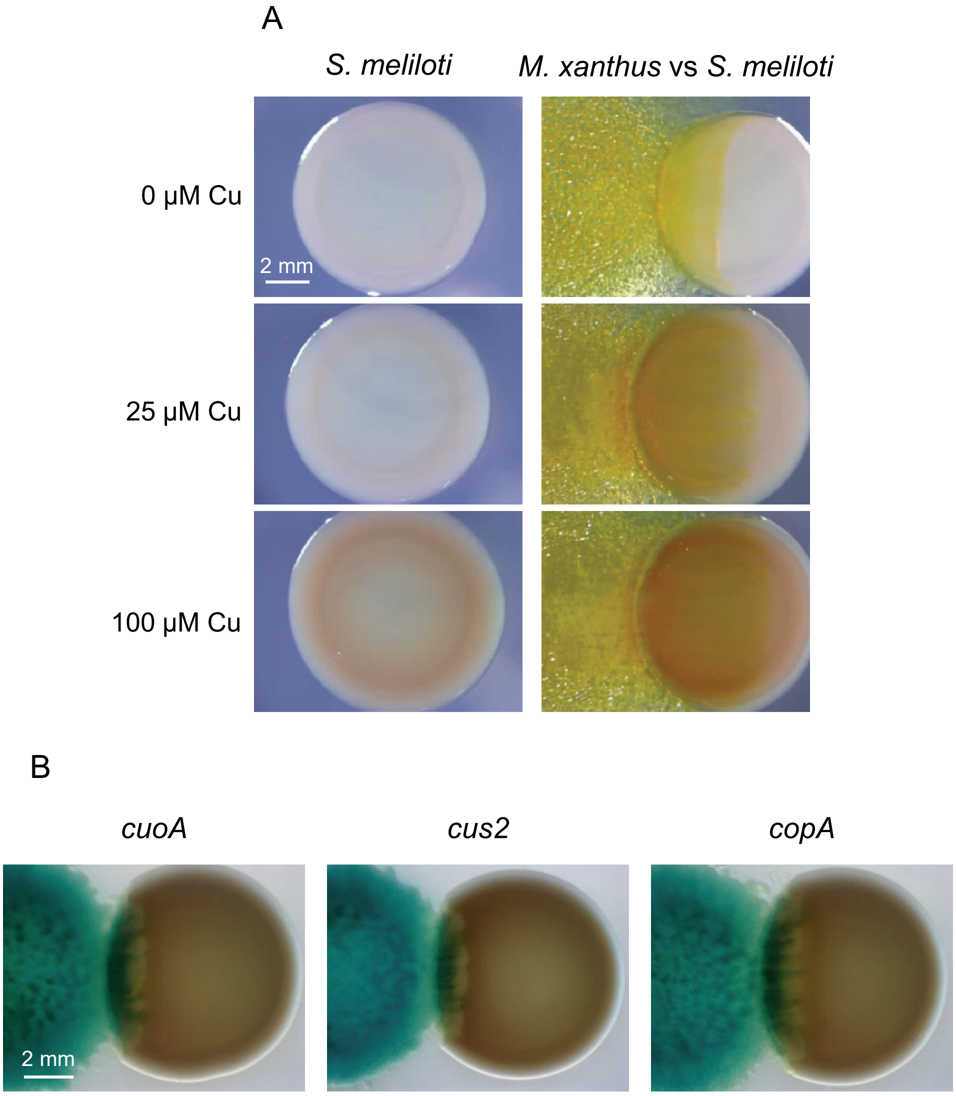

Figure S1. Predatory activity of *M. xanthus* versus *S. meliloti* GR4. **(A)** The wild-type strain of *M. xanthus* was co-cultured with *S. meliloti* GR4 in the presence of various copper concentrations and in the absence of this metal. Pictures were taken at 72 h of incubation under a dissecting microscope with illumination from the top of the colonies. **(B)** *M. xanthus* strains harboring fusions between promoters of genes *cuoA*, *cus2* and *copA*, and *lacZ* were co-cultured with *S. meliloti* GR4. Cells were spotted onto CTT agar plates containing 300 µM copper and 100 µg/ml X-gal to visualize the blue color development when the promoters are activated by the metal. Pictures were taken after 48 h of incubation under a dissecting microscope with illumination from the bottom of the colonies.
