# Supplementary material for: Copper and Melanin Play a Role in Myxococcus xanthus Predation on Sinorhizobium meliloti
Source: Front Microbiol. 2020 Feb 4;11:94. doi: 10.3389/fmicb.2020.00094 (PMC7010606; doi:10.3389/fmicb.2020.00094)
Supplement: Supplementary file 4 [file Image_2.pdf]

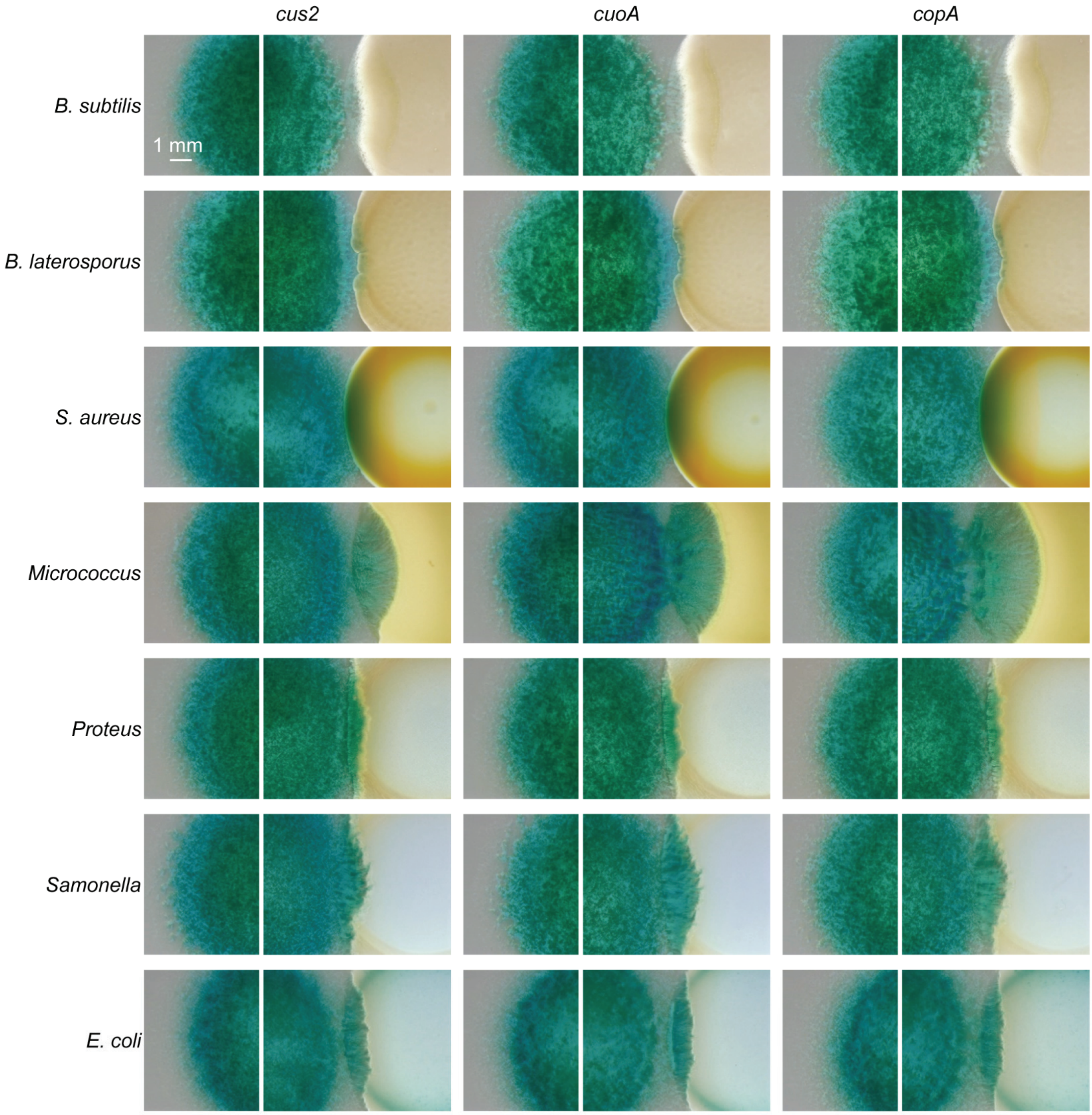

Figure S2. Detection of copper accumulation in the predatory interface of *M. xanthus* and several bacteria. *M. xanthus* strains harboring fusions between genes *cuoA*, *cus2* and *copA*, and *lacZ* were co-cultured with Gram-positive and Gram-negative bacteria in the presence of 300 μM of copper. Strains were spotted onto CTT agar plates containing 100 μg/ml X-gal to visualize the blue color development. Pictures were taken after 48 h of incubation under a dissecting microscope with illumination from the bottom of the colonies.
